# Five‐Year Experience of Haemophilia Centre Certification Performed by the German, Austrian and Swiss Society for Thrombosis and Haemostasis Research

**DOI:** 10.1111/hae.70193

**Published:** 2025-12-26

**Authors:** Hermann Eichler, Hagen Bönigk, Susan Halimeh, Christina Hart, Judith Hörner, Robert Klamroth, Ralf Knöfler, Christoph Königs, Thomas Lang, Florian Langer, Wolfgang Miesbach, Johannes Oldenburg, Ramona Oral, Martin Olivieri, Christian Pfrepper, Jan Pilch, Ute Scholz, Werner Streif, Manuela Albisetti

**Affiliations:** ^1^ Institute of Clinical Haemostaseology and Transfusion Medicine Saarland University and Saarland University Hospital Homburg Germany; ^2^ MVZ Limbach Magdeburg Magdeburg Germany; ^3^ Coagulation Center Rhein‐Ruhr Duisburg Germany; ^4^ Department of Hematology and Oncology University Hospital Regensburg Regensburg Germany; ^5^ ClarCert GmbH Neu‐Ulm Germany; ^6^ Internal Medicine Vivantes Clinic Friedrichshain Berlin Germany; ^7^ Faculty of Medicine and University Hospital Carl Gustav Carus Department of Paediatrics Paediatric Haemostaseology Unit, Technische Universität Dresden Dresden Germany; ^8^ Department of Paediatrics and Adolescent Medicine Goethe University Frankfurt University Hospital Frankfurt am Main Germany; ^9^ Gerinnungsambulanz Südheide Hohne Germany; ^10^ Medical Clinic II and Policlinic, Centre for Oncology University Medical Centre Eppendorf Hamburg Germany; ^11^ Medical Clinic II, Institute of Transfusion Medicine Goethe University Frankfurt am Main Germany; ^12^ Institute of Experimental Haematology and Transfusion Medicine University Clinic Bonn Bonn Germany; ^13^ Paediatric Thrombosis and Haemostasis Unit, Paediatric Haemophilia Centre Dr. Von Hauner Children's Hospital LMU Munich Germany; ^14^ Division of Haemostaseology, Department of Hematology, Cellular Therapy, Haemostaseology and Infectiology University Hospital Leipzig Leipzig Germany; ^15^ Institute for Transfusion Medicine and Haemostasis University Medical Centreaugsburg Augsburg Germany; ^16^ Centre of Coagulation Disorders Leipzig Germany; ^17^ Department of Paediatrics and Adolescent Medicine Medical University of Innsbruck (MUI) Innsbruck Austria; ^18^ Children's Research Centre and Division of Hematology University Children's Hospital Zurich Zurich Switzerland

## Abstract

**Introduction:**

To enhance the treatment quality in haemophilia centres (HC), the German, Austrian and Swiss Society for Thrombosis and Haemostasis Research (GTH) started in 2017 the development of guidelines to define the structural and process quality of HC, essentially related to the EUHANET guidelines.

**Aim:**

A certification process for HC located in Germany, Austria and Switzerland started in 2019.

**Methods:**

Within the first 5 years, 17 Haemophilia Comprehensive Care Centres (HCCC; ≥ 40 patients with severe HaemA/HaemB or vWD Type 3) and 6 Haemophilia Treatment Centres (HTC; ≥ 10 patients) were certified following a 1‐day on‐site audit performed by two trained haemophilia doctors. Experienced quality management staff supported the audit preparation in the HC. The entire certification process was coordinated by the accredited certification body ClarCert.

**Results:**

In the vast majority of the HCs, the precise specifications of the GTH guidelines in combination with the intensive audit preparation resulted in a mean number of only 2.80 nonconformities per audit (range 0–14). In addition, a mean number of 5.96 (range 2–13) recommendations were documented by the auditors. The feedback of the centres showed a high level of satisfaction with an excellent rating for both the professional auditors and ClarCert as the organizing certification body.

**Conclusion:**

After the successful implementation of the certification process, a certificate is now an essential prerequisite for HC in Germany to conclude separate contracts regarding comprehensive care with health insurance bodies.

## 1 Introduction

Since the 1970s, specialized haemophilia centres (HC) have been established for the complex and cost‐intensive treatment of patients with coagulation disorders. Their focus is on long‐term patient care by a multidisciplinary team of medical and non‐medical specialists who work together in a coordinated manner [1]. Treatment optimized in this way has been proven to increase both the life expectancy and quality of life of patients with haemophilia [2]. From 2005 to 2007, the European Principles for Haemophilia Treatment were developed by an international and interdisciplinary group of experts and presented to the European Parliament in 2009 [1]. Moreover, the European Commission, through an expert committee of the Department of Health and Food Safety, developed and published initial recommendations for the treatment of patients with rare diseases [3]. At the same time, independent projects were launched in several member states of the European Union to develop standards for the treatment of patients with rare coagulation disorders, on the basis of which national certification of HC could be achieved [4, 5, 6]. These experiences were incorporated into the European Guidelines for the Certification of Haemophilia Centres by the European Haemophilia Network (EUHANET) [7]. In 2017, and based on these EUHANET guidelines, the German, Austrian and Swiss Society for Thrombosis and Haemostasis Research (GTH) started to develop a guideline on the structural and process quality of HC in German‐speaking countries and published it in 2019, with a first revision in 2024 [8, 9 (Supplement)]. This guideline in the German language was intended to generate a transparent catalogue of criteria for defining the structural and process quality of HC, and to create a basis for the certification of HC under the scientific leadership of the GTH. This certification process for HC was established in 2019.

## 2 Methods

### 2.1 Structure of the GTH Certification Process

The GTH, as the guideline‐defining medical scientific society, cares for the content of the certification system, but not its implementation. Therefore, the management of the certification system was delegated to the German company ClarCert, an accredited and independent certification body active in various areas of medical quality assurance [10]. In this context, certification means the attestation of a system's conformity with defined requirements by an independent body. In keeping with the separation of powers, the bodies of the certification system are divided into the legislative branch, called the Certification Commission (CC), and the judicial branch, called the Certificate Issuing Committee (CiC). In addition, the executive branch is represented by the certification body ClarCert, which coordinates the pre‐ and post‐audit administrative issues, and expert haemophilia doctors as auditors. Each body is responsible for its assigned tasks in order to ensure an independent certification process. The members of the CC are appointed by the GTH board of directors, and their task is to further develop the certification criteria as well as to interpret and clarify individual requirements. In contrast, the CiC ensures the independence of the individual certification procedures and the final evaluation of the process. Before issuing, maintaining, or renewing a certificate, the CiC evaluates the required criteria based on the audit documentation. Thus, issuance of a certificate requires the approval of the CiC [10]. Finally, the certification body ClarCert cares for the overall coordination of the process and commissions the auditors. These are well‐experienced haemophilia doctors recruited by the GTH who have been extra trained in quality management issues to carry out on‐site audits at the applying HC.

### 2.2 Certification Procedure

**FIGURE 1:**
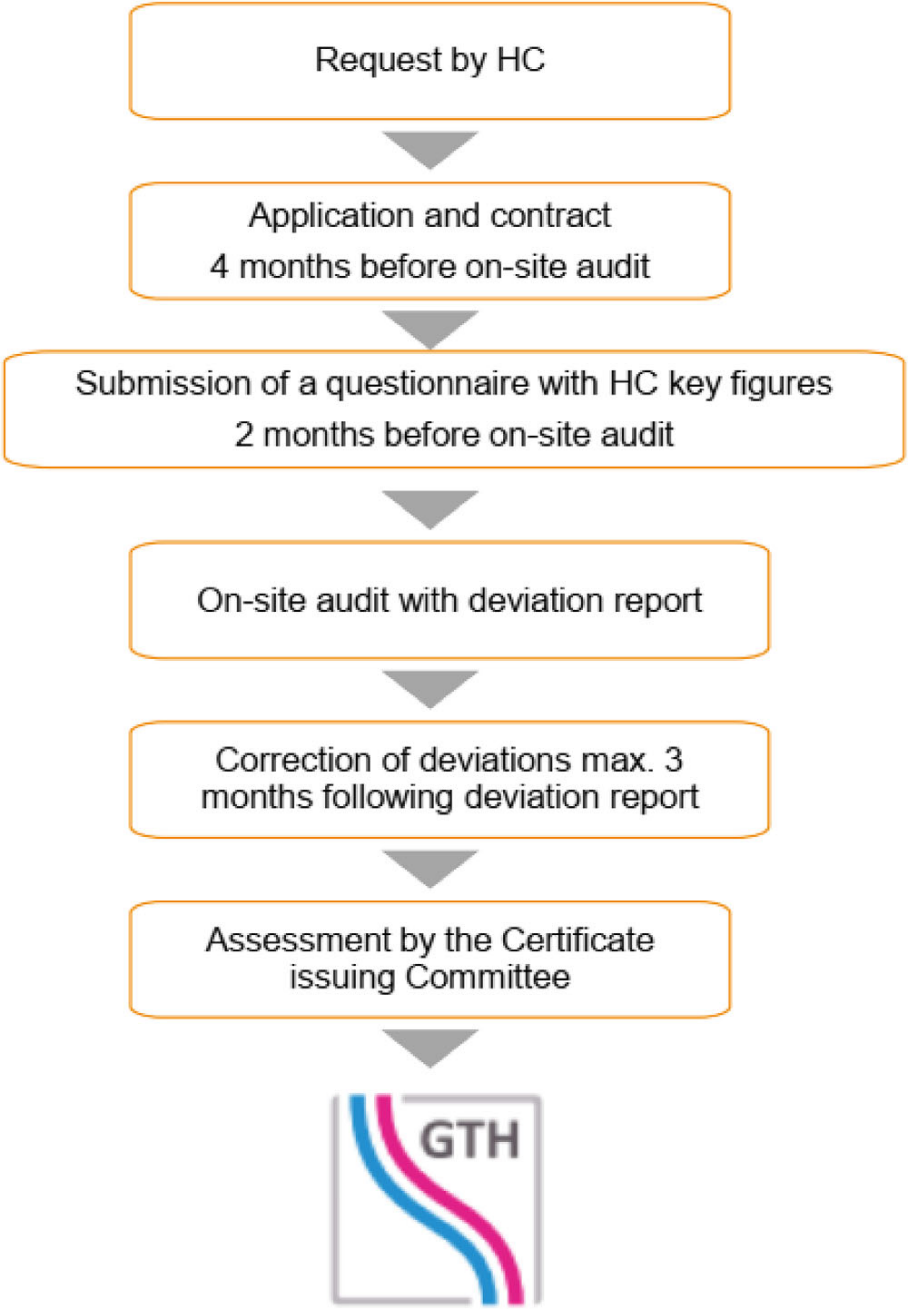
Flowchart giving an overview of the certification process.

The process starts with a formal request from the HC to ClarCert (Figure 1). This involves providing key information, including its requested classification as a Haemophilia Comprehensive Care Centre (HCCC; ≥ 40 patients with severe HaemA/HaemB or vWD Type 3) or as a Haemophilia Treatment Centre (HTC; ≥ 10 patients). ClarCert reviews this data to determine whether certification is generally possible. If so, a quote is prepared, and the centre submits a formal application to initiate the certification process. The contract with ClarCert includes that the costs for the certification process have to be covered by the applying HC. The initial certification cost by ClarCert is currently 3990 euros (US$4629, excluding auditor travel expenses). At best, contracting should be done 4 months before the desired audit date. The next step for ClarCert is to activate the audit team of two suitable haemophilia doctors, one as lead auditor. In parallel, the HC prepares the procedural documents. In particular, a point‐by‐point electronic questionnaire must be completed listing each single criterion of the GTH guideline and assessing whether the HC evaluates to meet or to fail the criteria. In terms of the quality management requirements, the GTH offers the applying centre support from well‐experienced quality management staff for the preparation of necessary quality management documentation. At least 2 months prior to the audit, the procedural documents are submitted to ClarCert for preliminary evaluation by the certification body and the lead auditor. This is followed by the preparation of the audit plan and, if necessary, further processing of the procedural documents by the HC. The 1‐day on‐site audit is carried out according to the audit plan. The auditors review the on‐site conditions for each individual item against the certification guideline. In cases of non‐compliance, a nonconformity is identified and reported to the centre's responsible personnel. Certification can generally only be granted if a nonconformity plan for eliminating all nonconformities is in place. In addition to nonconformities, the auditors can also address recommendations, which the centre may but is not required to consider. The audit concludes with a final result meeting with the HC personnel and the subsequent creation of an audit report. It documents nonconformities requiring correction but also provides suggestions for quality improvements. In the event of nonconformities, the HC has a maximum of 3 months to determine the corrective measures and to document the elimination to ClarCert. In the case of major nonconformities, a follow‐up audit can be conducted if necessary. The auditors evaluate the corrective measures initiated by the HC and, if positive, recommend granting certification. Finally, the CiC reviews all process documents and, if positive, issues a certificate valid for 5 years. Until recertification, the HC has to submit an updated electronic questionnaire to ClarCert on an annual basis to verify whether all certification criteria continue to be met. Recertification has to be carried out 5 years after the first‐time audit. To reissue the certificate, a recertification process is necessary at a cost of currently 3670 euros (US$4258, excluding auditor travel expenses).

## 3 Results

### 3.1 Timeline and Results of the Certification Process for Period 2019–2024

In 2019, as the first year of the process, ClarCert received four certification requests. In October 2019, the first application from a German university‐based HCCC was submitted to initiate the certification process. The initial on‐site audit took place in December 2019, with the issuance of the first certificate in February 2020. Table 1 shows the further chronological progression of the certification processes over the first 5 years with 25 performed on‐site audits (23 first‐time audits, one repeat audit, and one audit for re‐certification after 5 years). This resulted in a total of 23 certified German HCs by the end of 2024. Moreover, the first on‐site audit at an HC in Switzerland will be conducted in 2025.

**TABLE 1:** Timeline of the evolution of the certification process.

Year	2019	2020	2021	2022	2023	2024
Request	4	8	6	6	5	2
Application for HCCC	1	5	3	3	4	1
Application for HTC	—	2	3	1	—	—
First‐time on‐site audit	1	4	8	4	5	1
Repeat audit for first‐time certification	—	—	1	—	—	—
On‐site audit for re‐certification	—	—	—	—	—	1
Number of issued certificates (cumulative)	—	1	10	17	20	23

The number of identified nonconformities and given recommendations in terms of the different certification topics are shown in Tables 2 and 3, respectively. Overall, a mean number of 2.80 (range 0–14) nonconformities and a mean number of 5.96 (range 2–13) recommendations per audit were documented. The number of nonconformities was highest in the four audits in 2020 focusing on the basic structure of the HC and quality management requirements but much less on issues regarding haemophilia treatment itself. The frequency of nonconformities decreased to an annual mean number of only 1.22 per audit or even below in the period 2021 to 2024. This decreasing number of nonconformities may be due to an improved audit preparation through support of the centres by QM‐experienced staff offered by the GTH. In contrast, the number of recommendations was on a higher level and quite stable over time, reflecting an intensive professional exchange of ideas between the auditors and the local haemophilia doctors during the on‐site audits.

**TABLE 2:** Type and number of nonconformities detected at on‐site audits.

Year	2019	2020	2021	2022	2023	2024
**Total number of on‐site audits**	**1**	**4**	**9**	**4**	**5**	**2**
Basic structure of the HC: number and qualifications of personnel; access to treatment documentation; time required for lab results	0	26	5	0	1	0
Compliance with quality management requirements; network of the multidisciplinary treatment team; active participation in registers	0	22	5	0	1	0
Diagnosis and treatment of coagulation disorders, including planning and implementation of surgical interventions	0	5	1	1	0	0
Information, education and training of patients and relatives	0	3	0	0	0	0
**Sum number of nonconformities**	**0**	**56**	**11**	**1**	**2**	**0**
**Mean nonconformities per on‐site audit**	**0**	**14.0**	**1.22**	**0.25**	**0.40**	**0**

**TABLE 3:** Type and number of recommendations given at on‐site audits.

Year	2019	2020	2021	2022	2023	2024
**Total number of on‐site audits**	**1**	**4**	**9**	**4**	**5**	**2**
Basic structure of the HC: number and qualifications of personnel; access to treatment documentation; time required for lab results	3	15	23	5	6	2
Compliance with quality management requirements; network of the multidisciplinary treatment team; active participation in registers	5	12	16	3	0	3
Diagnosis and treatment of coagulation disorders, including planning and implementation of surgical interventions	3	16	14	6	4	6
Information, education and training of patients and relatives	2	2	2	1	0	0
**Sum number of recommendations**	**13**	**45**	**55**	**15**	**10**	**11**
**Mean recommendations per on‐site audit**	**13.0**	**11.25**	**6.11**	**3.75**	**2.0**	**5.5**

After the successful completion of the certification, all centres were asked for their feedback regarding the performance and competence of the auditors as well as the quality of the certification body ClarCert, with 12 centres (52.2%) returning the respective questionnaire. Table 4 contains the mean feedback results for the different items with possible ratings between 1 (excellent) and 4 (poor). As there was no mean grade above 1.25, the performance of both the auditors and ClarCert was rated as excellent.

**TABLE 4:** Survey results from the audited HC regarding the competence of the auditors and the performance of the certification body ClarCert (*N* = 12; mean values).

Preparation and planning of the on‐site audit	
Preparation for the audit based on the electronic questionnaire	1.00
Participation in the design of the audit planning	1.24
Availability of auditors	1.07

Experience during the on‐site audit	
Professional expertise of the auditors	1.07
Open‐minded and attentive behaviour of the auditors during the audit	1.00
Questions were orientated towards the requirements of the certification	1.24
Questions were asked in an appropriate manner	1.00
Auditors created a trusting atmosphere in all audit phases	1.00
There was a clear designation of positive impressions	1.07
There was a clear designation of recommendations and recommendations	1.07
In the concluding meeting, the audit results were presented appropriately	1.19
The evaluation was based on actual facts	1.07
The audit report provided a complete and accurate account of the audit results	1.15

Evaluation of the certification body ClarCert	
Availability of the employees	1.00
Professional expertise	1.00
Process and control of the certification procedure	1.00

*Note*: Possible ratings: 1 = excellent; 2 = good; 3 = moderate; 4 = poor.

## 4 Discussion

Specialized medical certification processes for quality‐assured care of complex and multi‐professional services have meanwhile found widespread application, for example, in the areas of blood stem cell transplantation or endoprosthetic surgery [11, 12]. Essential components of such certifications are on‐site audits by independent experts to assess compliance with predefined quality standards [11, 12]. The longest experience in conducting quality audits in the field of haemophilia is held by the UK Haemophilia Doctors Organisation, which started its programme for the certification of HC in 1993 [4]. In 2012, results of the 3‐yearly audit assessment of 22 Comprehensive Care HC were published [4]. By analysing the 2009 audit reports, 122 issues were raised in total (median 6 per centre, range 1–11). Nevertheless, it is stated that since the implementation of the audit process, the recommendations have resulted in major quality improvements in UK HCCC. Moreover, the audit process was considered to be highly effective and recommended as a model for comparable projects in other countries [4].

From 2012 to 2015, EUHANET guided a project co‐funded by the European Commission to implement an HC certification scheme [13]. Centres could apply for certification as either a European Haemophilia Treatment Centre or a European HCCC depending on key areas such as the number of patients registered or services offered. The 115 applications from 30 European countries were assessed by a committee only on the basis of questionnaires filled out by the centres, but time‐consuming on‐site audits were not performed in this project. As the next step and based on these experiences, the European Association of Haemophilia and Allied Disorders (EAHAD) established an Accreditation Working Group to update the European guidelines for certification of HC, and a pilot project for the accreditation of HC, including on‐site audit, has been designed [14].

In 2019 and 2024, the GTH published its guideline on structural and process quality of HC in the German language and essentially related the EUHANET and EAHAD guidelines [7, 8, 9]. On this basis and under the GTH leadership, a certification process including the performance of on‐site audits was implemented in the German‐speaking countries Austria, Switzerland and Germany. As a consequence of the experiences in other countries, the GTH board decided to focus on defining the technical criteria and on recruiting the expert auditors, while the complete administration of the certification process was outsourced to an accredited professional certification body. As management of haemophilia is multidisciplinary, a certification process of HC needs to include all specific issues, such as physiotherapy, nursing, or psychosocial aspects of haemophilia care, as the GTH certification procedure does. In terms of the audit team composition, this would consequently mean that for each audit a large team needs to be recruited to cover all the different treatment aspects by an expert. On the other hand, such an approach is timely, costly and difficult to organize. Therefore, the GTH decided to minimize administrative effort and cost by defining a small audit team of only two experienced haemophilia doctors, but not to include other members of the multi‐professional team—at least for the starting phase of the project. However, the doctors assess the aspects that would have been reviewed by a multidisciplinary audit team. With these specifications, it was possible to carry out 23 certification procedures within 5 years by a team of only 10 engaged auditors. The audited centres and the auditors had the opportunity for an intensive professional exchange, from which both sides benefited. The administration of the process by ClarCert led to a significant reduction in the workload for the auditors, for example, because ClarCert carried out the initial review of the documents submitted by the centres. In addition, the certification organization ensured that the greatest possible transparency and independence could be achieved. This resulted in a high level of satisfaction among the applying centres regarding the certification process.

## 5 Conclusion

Since 2017 and under the leadership of the GTH, the development of a quality guideline for HC followed by the implementation of a certification process was successfully established. On‐site audits were conducted by an extra‐trained team of haemophilia doctors. Between 2019 and 2024, 25 audits were performed, and 23 HCs successfully completed the certification process. Meanwhile, a certificate is now an essential prerequisite for HC in Germany to conclude separate contracts regarding comprehensive care with health insurance bodies.

## Author Contributions

All authors contributed equally to its content.

## Funding

The authors have nothing to report.

## Ethics Statement

For this study, only data from a certification process were retrospectively analysed. No patient data were evaluated. Therefore, approval from an ethics committee was not required.

## Conflicts of Interest

On behalf of all co‐authors, the first author declares no conflicts of interest regarding this publication.

## Data Availability

Data sharing is not applicable to this article, as no new data were created or analysed in this study.
